# Supplementary figures and images for: CT-derived body composition: Differential association with disease, age and inflammation in a retrospective cohort study
Source: PLoS One. 2024 Mar 21;19(3):e0300038. doi: 10.1371/journal.pone.0300038 (PMC10956827; doi:10.1371/journal.pone.0300038)

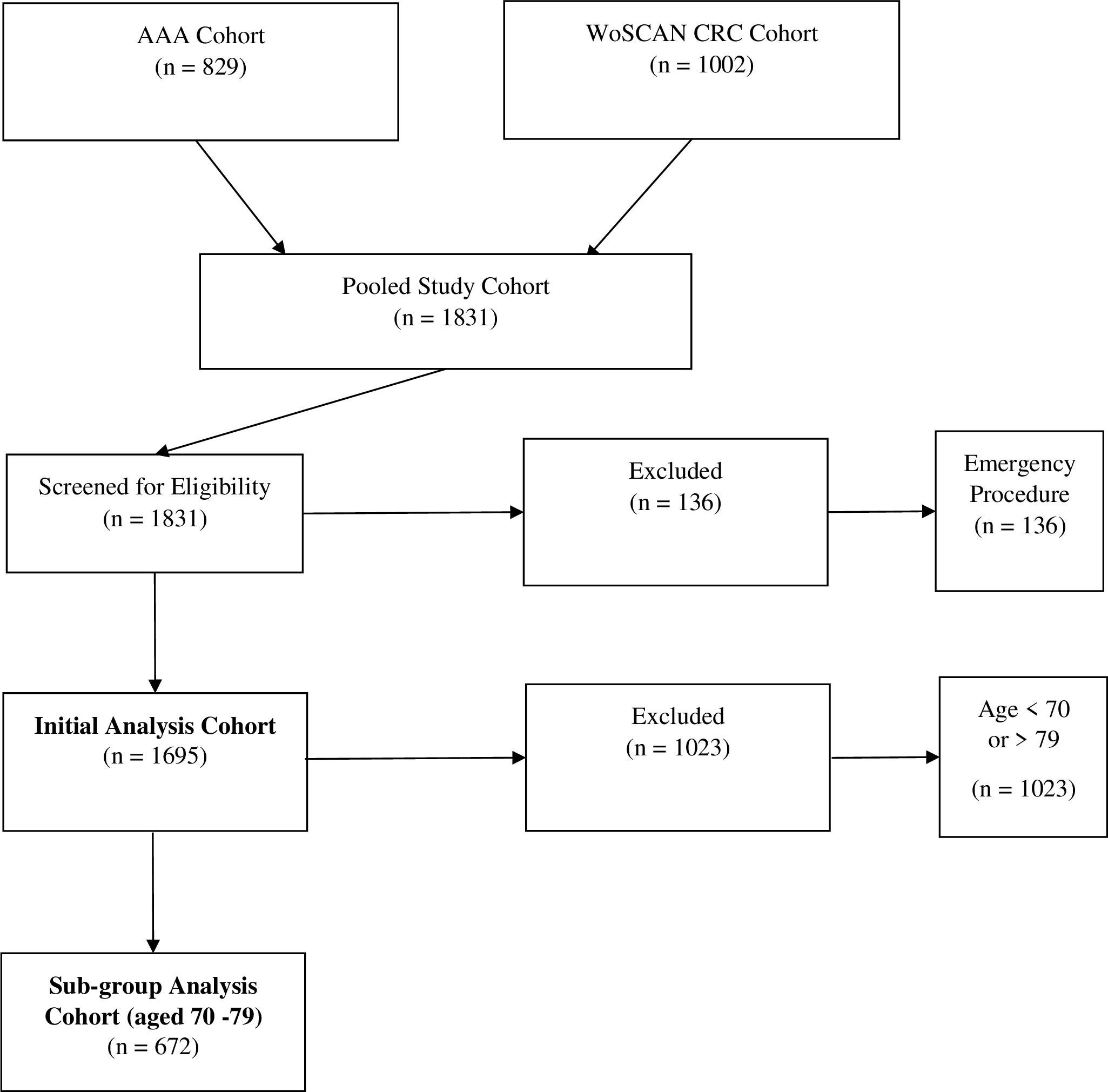

Supplement: S1 Fig — AAA: Abdominal aortic aneurysm. CRC: Colorectal cancer. WoSCAN: West of Scotland Cancer Network. (TIF) [file pone.0300038.s001.tif]
