# Supplementary material for: CT-derived body composition: Differential association with disease, age and inflammation in a retrospective cohort study
Source: PLoS One. 2024 Mar 21;19(3):e0300038. doi: 10.1371/journal.pone.0300038 (PMC10956827; doi:10.1371/journal.pone.0300038)
Supplement: S1 Table — (DOCX) [file pone.0300038.s002.docx]

| **S1 Table: The calculation of inflammation-based prognostic scores using pre-operative blood results.** | | |
| --- | --- | --- |
| NLR | Absolute Neutrophil Count / Absolute Lymphocyte Count | |
|  | | |
| mGPS | mGPS 0 | CRP ≤ 10mg/L |
|  | mGPS 1 | CRP > 10mg/L and Albumin > 35 g/L |
|  | mGPS 2 | CRP > 10mg/L and Albumin < 35 g/L |
|  | | |
| SIG | SIG 0 | mGPS 0 and NLR < 3 |
|  | SIG 1 | mGPS 0 and NLR 3 – 5  or  mGPS 1 and NLR < 3 |
|  | SIG 2 | mGPS 0 and NLR > 5  or  mGPS 2 and NLR < 3  or  mGPS 1 and NLR 3 - 5 |
|  | SIG 3 | mGPS 1 and NLR > 5  or  mGPS 2 and NLR 3 - 5 |
|  | SIG 4 | mGPS 2 and NLR > 5 |
| NLR: Neutrophil:Lymphocyte Ratio. mGPS: Modified Glasgow Prognostic Score (mGPS). SIG: Systemic Inflammatory Grade. CRP: C-reactive protein. | | |
