# Supplementary material for: CT-derived body composition: Differential association with disease, age and inflammation in a retrospective cohort study
Source: PLoS One. 2024 Mar 21;19(3):e0300038. doi: 10.1371/journal.pone.0300038 (PMC10956827; doi:10.1371/journal.pone.0300038)
Supplement: S2 Table — (DOCX) [file pone.0300038.s003.docx]

**S2 Table: Classification of abnormal CT-derived body composition**

| **High visceral fat area** | | |
| --- | --- | --- |
| Author: Doyle et al[27] | Male | >160cm^2^ |
|  | Female | >80cm^2^ |
| **High subcutaneous fat index** | | |
| Author: Ebadi et al[26] | Male | >50cm^2^/m^2^ |
|  | Female | >42cm^2^/m^2^ |
| **Low skeletal muscle index** | | |
| Author: Martin et al[25] | Male, BMI ≤ 25 kg/m^2^ | <43cm^2^/m^2^ |
|  | Male, BMI > 25 kg/m^2^ | <53cm^2^/m^2^ |
|  | Female | <41cm^2^/m^2^ |
| **Low skeletal muscle density** | | |
| Author: Martin et al[25] | BMI ≤ 25 kg/m^2^ | <41 HU |
|  | BMI > 25 kg/m^2^ | <33 HU |
| HU: Hounsfield units. BMI: body mass index. | | |
