# Supplementary material for: CT-derived body composition: Differential association with disease, age and inflammation in a retrospective cohort study
Source: PLoS One. 2024 Mar 21;19(3):e0300038. doi: 10.1371/journal.pone.0300038 (PMC10956827; doi:10.1371/journal.pone.0300038)
Supplement: Attachment — Submitted filename: AAA vs. CRC. Response to reviewers.docx [file pone.0300038.s004.docx]

24^th^ January 2024

Ref: PONE-D-23-29602
"CT-derived body composition: differential association with disease, age and inflammation in a retrospective cohort study.”

Dear Editor(s),

The authors are grateful for the opportunity to respond to the reviewers’ comments. We have considered them carefully and these have improved the manuscript. Please find our response to the comments below.

Editor’s Comments:

*1. Please ensure that your manuscript meets PLOS ONE's style requirements, including those for file naming. The PLOS ONE style templates can be found at*

[*https://journals.plos.org/plosone/s/file?id=wjVg/PLOSOne_formatting_sample_main_body.pdf*](https://journals.plos.org/plosone/s/file?id=wjVg/PLOSOne_formatting_sample_main_body.pdf) *and* 
[*https://journals.plos.org/plosone/s/file?id=ba62/PLOSOne_formatting_sample_title_authors_affiliations.pdf*](https://journals.plos.org/plosone/s/file?id=ba62/PLOSOne_formatting_sample_title_authors_affiliations.pdf)

Comment noted – we have addressed the style requirements throughout the revised manuscript.

*2. In this instance it seems there may be acceptable restrictions in place that prevent the public sharing of your minimal data. However, in line with our goal of ensuring long-term data availability to all interested researchers, PLOS’ Data Policy states that authors cannot be the sole named individuals responsible for ensuring data access (http://journals.plos.org/plosone/s/data-availability#loc-acceptable-data-sharing-methods).*

*Data requests to a non-author institutional point of contact, such as a data access or ethics committee, helps guarantee long term stability and availability of data. Providing interested researchers with a durable point of contact ensures data will be accessible even if an author changes email addresses, institutions, or becomes unavailable to answer requests.*

*Before we proceed with your manuscript, please also provide non-author contact information (phone/email/hyperlink) for a data access committee, ethics committee, or other institutional body to which data requests may be sent. If no institutional body is available to respond to requests for your minimal data, please consider if there any institutional representatives who did not collaborate in the study, and are not listed as authors on the manuscript, who would be able to hold the data and respond to external requests for data access? If so, please provide their contact information (i.e., email address). Please also provide details on how you will ensure persistent or long-term data storage and availability.*

Comment noted – the data presented in this manuscript will be stored in the University of Glasgow “Enlighten: Research Data” repository in line with institutional guidelines for data storage and retention. Details for accessing these data can be found at: https://www.gla.ac.uk/myglasgow/openresearch/researchdatamanagement/enlightenresearchdatainformation/

*3. Please include captions for your Supporting Information files at the end of your manuscript, and update any in-text citations to match accordingly. Please see our Supporting Information guidelines for more information:* [*http://journals.plos.org/plosone/s/supporting-information*](http://journals.plos.org/plosone/s/supporting-information)*.*

Comment noted – these have been addressed in the revised submission.

*4. We note that there is identifying data in the Supporting Information file <Supplemental Table 2.docx>. Due to the inclusion of these potentially identifying data, we have removed this file from your file inventory. Prior to sharing human research participant data, authors should consult with an ethics committee to ensure data are shared in accordance with participant consent and all applicable local laws.*

*Data sharing should never compromise participant privacy. It is therefore not appropriate to publicly share personally identifiable data on human research participants. The following are examples of data that should not be shared:*

*-Name, initials, physical address
-Ages more specific than whole numbers
-Internet protocol (IP) address
-Specific dates (birth dates, death dates, examination dates, etc.)
-Contact information such as phone number or email address
-Location data
-ID numbers that seem specific (long numbers, include initials, titled “Hospital ID”) rather than random (small numbers in numerical order)*

*Data that are not directly identifying may also be inappropriate to share, as in combination they can become identifying. For example, data collected from a small group of participants, vulnerable populations, or private groups should not be shared if they involve indirect identifiers (such as sex, ethnicity, location, etc.) that may risk the identification of study participants.*

*Additional guidance on preparing raw data for publication can be found in our Data Policy (*[*https://journals.plos.org/plosone/s/data-availability#loc-human-research-participant-data-and-other-sensitive-data*](https://journals.plos.org/plosone/s/data-availability#loc-human-research-participant-data-and-other-sensitive-data)*) and in the following article:* [*http://www.bmj.com/content/340/bmj.c181.long*](http://www.bmj.com/content/340/bmj.c181.long)*.*

*Please remove or anonymize all personal information (name), ensure that the data shared are in accordance with participant consent, and re-upload a fully anonymized data set. Please note that spreadsheet columns with personal information must be removed and not hidden as all hidden columns will appear in the published file.*

Comment noted – this appears to have been a miscommunication on our part. The names listed in supplemental table 2 do not pertain to identifiable information for study participants, in fact these are the surnames of the first authors of the published papers from which thresholds of body composition parameters were taken. Supplemental table 2 has been revised (the word “author” and the suffix “et al”) added to ensure that this is clear in the revised submission (see below, changes highlighted):

| **High visceral fat area** | | |
| --- | --- | --- |
| Author: Doyle et al[27] | Male | >160cm^2^ |
|  | Female | >80cm^2^ |
| **High subcutaneous fat index** | | |
| Author: Ebadi et al[26] | Male | >50cm^2^/m^2^ |
|  | Female | >42cm^2^/m^2^ |
| **Low skeletal muscle index** | | |
| Author: Martin et al[25] | Male, BMI ≤ 25 kg/m^2^ | <43cm^2^/m^2^ |
|  | Male, BMI > 25 kg/m^2^ | <53cm^2^/m^2^ |
|  | Female | <41cm^2^/m^2^ |
| **Low skeletal muscle density** | | |
| Author: Martin et al[25] | BMI ≤ 25 kg/m^2^ | <41 HU |
|  | BMI > 25 kg/m^2^ | <33 HU |
| HU: Hounsfield units. BMI: body mass index. | | |

*5. Please review your reference list to ensure that it is complete and correct. If you have cited papers that have been retracted, please include the rationale for doing so in the manuscript text, or remove these references and replace them with relevant current references. Any changes to the reference list should be mentioned in the rebuttal letter that accompanies your revised manuscript. If you need to cite a retracted article, indicate the article’s retracted status in the References list and also include a citation and full reference for the retraction notice.*

Comment noted – the reference list has been reviewed and is correct.

Reviewer 1 Comments:

*In this interesting paper, the Authors compare skeletal muscle quantity and quality reduction in AAA and CRC patients, and evaluate if systemic inflammation influence SMI and SMD reduction. The work is correctly conducted, the rationale is clear and the paper clearly written.*

The authors acknowledge these supportive comments of the reviewer.

*I have only some minor suggestions:
-introduction: "Low SMD appears to be associated with intramuscular lipid content[11], and may represent poor quality muscle through replacement of healthy muscle tissue with adipose tissue.". I think that SMD represents fat inside the muscle fibers (while intermuscolar adipose tissue area or IMAT represents adipose tissue infiltrating the muscle, so within fibers and beyond the fascia), so probably low SMD does not exactly represent replacement of muscle with adipose tissue, but instead the accumulation of fat droplets within muscle cells. Of course the poor effect on muscle quality is the same.*

Point taken – this sentence has been revised as below (Page 4, Lines 80 - 83):

“Low SMD appears to be associated with increased lipid content within myocytes[11], and may therefore represent poor quality muscle through accumulation of fat droplets in otherwise healthy muscle tissue.”

*Methods: it's not clear how the SMD values were made homogeneous between the two cohorts. "in order to mitigate the potential risk of bias in SMD readings between these 2 phases SMD analyses were repeated using values in the AAA cohort transformed as per Rollins et al". First, Rollins et al analyzed a small cohort and even if here the Authors use the equations proposed by Rollins et al, the use of two different phases is still a potential source of bias. Second, how was the equation used? Did the Authors obtain portal venous phase SMD values also on the AAA cohort by using the equation? or they obtain unenhanced SMD for the whole cohort? If the cut-off used for low SMD definition is that from Martin et al., it was obtained on unenhanced CT, hence defining low SMD on enhanced CT by using Martin's cut-off can introduce misclassification. Maybe using SMD as a continuous variable and reporting also mean and SD in different groups can be useful?*

Point taken – this has been addressed as below:

Methods (Page 4, Lines 159 - 161):

“…in order to mitigate the potential risk of bias in SMD readings between these 2 phases SMD analyses were repeated using values in the AAA cohort transformed from arterial phase to comparable portal venous phase as per Rollins *et al* (SMD_Rollins_)[28].”

AND

Methods (Page 4, Lines 168 - 169):

“Differences between continuous variables were assessed using the Mann-Whitney U Test, with median (interquartile range, IQR) reported.”

AND

Results (Page 18, Lines 273 - 278):

“When the SMD analyses were repeated using SMD_Rollins_, low SMD was less prevalent in the AAA cohort (21% vs. 66%, *p* < 0.001). To account for potential categorical misclassification based on phase of scan, continuous values of SMD and SMD_Rollins_ were analysed. Median (IQR) SMD in the AAA vs. CRC cohorts was 37.8 (9.5) HU vs. 31.8 (12.7) HU (*p* < 0.001). Median (IQR) SMD_Rollins_ in the AAA vs. CRC cohorts was 40.2 (9.3) HU vs. 31.8 (12.7) HU (*p* < 0.001).”

AND

Limitations (page 21, Lines 355 – 359):

“Rollins et al report that, as compared to the portal venous phase, arterial phase CTs may underestimate SMD values[28]. Given the present study observed lower SMD in the CRC cohort (portal venous phase) as compared to the AAA cohort (arterial phase) it is therefore likely that the effect of phase is underestimating rather than overestimating the strength of our observations.”

Reviewer 2 Comments:

*I read with extreme interest the article by Bradley et al. on CT-derived body composition and its differential association with disease, age, and inflammation. Although this is a retrospective cohort study, it sheds attractive light on prognosis and body habitus in two chronic disease settings, the oncologic (colon) and the vascular (aortic aneurysm) settings. The message is that aging and systemic inflammation are ubiquitous determinants of loss of muscle mass and content, independently of disease setting. The paper is well written.*

The authors acknowledge these supportive comments of the reviewer.

*I only have some concerns inherent in the possible association with broad-spectrum anti-inflammatory pharmacological therapies (e.g., in cancer, steroids, aortic aneurysms, statins).*

Point taken – this is a pertinent point however unfortunately these data are not available for the majority of the cohort, and this has been addressed as below:

Limitations (page 21, Lines 362 – 368):

“The potentially confounding effect of pharmacological therapies such as steroids, chemotherapy, statins on muscle tissue and the systemic inflammatory response may be considered. These data were not available for the present study cohorts, and is therefore a potential source of bias. However, typical UK practice does not advocate neoadjuvant steroid or chemotherapy for colonic cancer, therefore any effect is likely to be small. The association between statins and the results of interest in the present study would be an important area for further research. Nevertheless, it is clear from the present work that aging and systemic inflammation are important determinants of loss of muscle mass and content, independent of disease setting.”

*Do the authors have bioumoral data such as troponin or CPK available? These indicators are associated with chronic vascular and cancer damage and could shed additional helpful light in the context.*

Point taken – unfortunately due to the retrospective nature of the cohort, blood tests which did not constitute routine care at the time of surgery are not available.

*How was the possible difference in dietary habits between the two populations considered?*

Point taken – this is an important potential confounding factor and accurate reporting of dietary habits is notoriously challenging to retrospectively record; this has been discussed as below:

Limitations (page 22, Lines 369 – 375):

“In the present study nutritional status may have been a potential confounding factor, however, the patient groups studied (i.e. patients undergoing intervention for AAA and patients undergoing surgery for colorectal cancer) are not recognised to be at high nutritional risk. For example, Chaar and colleagues reported that in patients undergoing intervention for AAA the mean (SE) pre-operative Nutritonal Risk Index was 103.6 (1.4); with values less than 100 conferring malnutrition[42]. Similarly, in a cohort of 363 patients undergoing surgery for CRC, 79% were classed as low risk of malnutrition based on MUST score[43].”

We greatly appreciate your continued consideration of this manuscript.

Yours sincerely,

Nicholas A Bradley

On behalf of the authors.
